# Comparison of two curing protocols during adhesive cementation: can the step luting technique supersede the traditional one?

**DOI:** 10.1007/s10266-020-00558-0

**Published:** 2020-10-31

**Authors:** Vincenzo Tosco, Riccardo Monterubbianesi, Giulia Orilisi, Simona Sabbatini, Carla Conti, Mutlu Özcan, Angelo Putignano, Giovanna Orsini

**Affiliations:** 1 Department of Clinical Sciences and Stomatology, Polytechnic University of Marche, Ancona, Italy; 2 Department of Materials, Environmental Science and Urban Planning, Polytechnic University of Marche, Ancona, Italy; 3 Division of Dental Biomaterials, Clinic for Reconstructive Dentistry, Center of Dental Medicine, University of Zurich, Zurich, ZRH, Switzerland

**Keywords:** Curing protocols, Resin luting agents, Degree of conversion, Kinetics of conversion degree, NIR spectroscopy

## Abstract

This study aims to compare the degree of conversion of two different curing protocols used during adhesive cementation. The following resin luting agents were tested: Hri Flow (MF) and pre-heated Hri Micerium (MH); light-cure Nexus Third Generation (NX3L) and dual-cure Nexus Third Generation (NX3D); dual cured RelyX Ultimate (RXU) and light-cure RelyX Veneers (RXL). For each tested material, ten samples were prepared and divided into two groups which had different curing protocols (P1 and P2): in P1, samples were cured for 40 s; in P2, samples were cured for 5 s, and then, after 20 s, cured again for additional 40 s. The degree of conversion (DC) was evaluated both during the first 5 min of the curing phase and after 1, 2, 7, 14 and 28 days (*p* = 0.05). Different trends were observed in DC values after 5 min by comparing P1 and P2. In both P1 and P2, DC decreased as follows, MH > MF > NX3L > RXL > RXU > NX3D. There were significant differences of DC values among all resin luting agents (*p* < 0.05) in P1, while no significant differences existed between MH and MF, and NX3L and RXL in P2. At 1, 2, 7, 14 and 28 days the light curing luting agents had a higher DC than the dual luting agents (*p* < 0.05). P1 and P2 were not statistically different at each time point (*p* > 0.05). Both P1 and P2 protocols let achieve an acceptable DC after 28 days. The tested P2 can be safely used to lute indirect restorations, simplifying the removal of cement excesses.

## Introduction

The development of reliable adhesive systems between the tooth and resin-based materials (RBM) has led to more conservative dental treatments. Before the introduction of modern adhesive materials, dentists prepared teeth to have mechanical retention, and in doing so, often sacrificed healthy dental tissue. The age of adhesive cementation has led to minimally invasive dentistry, in which the clinician can lute indirect restorations using resin luting agent thereby preserving dental tissue.

Resin luting agents are intermediates between the tooth substrate, with or without a bonding agent, and the indirect restoration. They can be divided into two categories: traditional resin-based composites and resin cements. These latter can be divided into light, self or dual cured according to the curing procedure, depending on own monomers and formulation.

A free radical reaction allows resin luting agents to move from a viscous to a rigid state in a process called polymerization. During the curing process, the terminal aliphatic C = C bonds are broken and converted into primary C–C covalent bonds between methacrylate monomers, the ratio of this conversion is described with the degree of conversion (DC). However, the formation of free radical varies with the activator system [1].

Multiple factors can influence the DC of luting resin agents such as their monomer content, the components of the activation system, and the type of polymerization. Moreover, the level of DC achieved during the polymerization directly influences the physical and mechanical properties of resin luting agents, as the whole RBM [2], therefore, affects the longevity of the indirect restoration [3]. Inferior mechanical properties, greater discoloration and degradation are the main drawbacks of a low DC, resulting in a resin luting agent with poor wear resistance and poor colour stability [4, 5].

Because during clinical application, the thickness of the indirect restoration can reduce the amount of curing light that reaches the resin luting agent, then the type of curing becomes a fundamental factor for the success of a restoration [6, 7]. For example, with a thick inlay/overlay restoration or with deep cavities, clinicians are uncertain whether the resin luting agent have been properly polymerized. Moreover, removing the excesses of resin luting agents could be difficult and time-consuming, mainly in the interdental space, because their hardening.

For this reason, during the luting phase, some clinicians adopt a step curing technique to fix the indirect restoration allowing the clinician to remove the soft excess of material around the indirect restoration. There are no scientific articles or evidences about the chemical stability after this kind of “step luting” procedure. Then, improving the knowledge on polymerization kinetics could be relevant, particularly during the cementation of an indirect restoration.

The aim of this study was to analyse the effects caused by two different curing protocols (P1 and P2) on the polymerization of various resin luting agents. The two null hypotheses were: 1) All the tested materials have the same DC; 2) The curing protocols do not affect the DC of tested materials.

## Materials and methods

### Samples preparation and FT-NIR analysis

The following resin luting agents were investigated: the light-cure flow resin composite, Enamel Plus HRi Flow, shade UD3 (MF) (Micerium, Avegno, Genova, Italy); the pre-heated light-cure high viscosity resin composite, Enamel Plus HRi, shade UD3 (MH) (Micerium, Avegno, Genova, Italy); the light-cure resin cement, Nexus Third Generation, shade Yellow (NX3L) (Kerr, Orange, CA, USA); the dual-cure resin cement, Nexus Third Generation, shade Yellow (NX3D) (Kerr, Orange, CA, USA); the light-cure resin cement, RelyX Veneer, shade A3 (RXL) (3M ESPE, St. Paul, MN), and the dual-cure resin cement, RelyX Ultimate, shade A3 (RXU) (3M ESPE, St. Paul, MN). The pre-heated composite MH was obtained by heating a compule for 10 min at 55 °C in the oven (Ena Heat, Micerium, Avegno, Genova, Italy), following the manufacturer instruction. The composition of the tested materials is described in Table 1.

**Table 1:** Composition of resin luting agents

Code	Brand (Shade)	Manufacturer	Type	Composition	Filler composition
MF	Enamel Plus HRi Flow (UD3)	Micerium, Avegno, Genova, Italy	Light-Cure flow resin composite	BisGMA, BDDMA, UDMA, glass filler highly dispersed SiO_2_	77% wt Unknown%vol
MH	Enamel Plus HRi (UD3)	Micerium, Avegno, Genova, Italy	Pre-heated Light cure resin composite	DiUDMA, BisGMA, BDDMA Filler: Particles of zirconium oxide and glass	80 wt% 63 vol%
NX3L	Nexus Third Generation (Yellow)	Kerr, Orange, CA, USA	Light-Cure resin cement	Uncured Methacrylate Ester Monomers, minors filler, pigments, radiopaque agent 20–40%	63 wt % 38 vol%
NX3D	Nexus Third Generation (Yellow)	Kerr, Orange, CA, USA	Dual-cure resin cement	Uncured Methacrylate Ester Monomers 20–40%	Unknown%wt 47 vol%
RXL	RelyX Veneer (A3)	3M ESPE, St.Paul, MN	Light-Cure resin cement	TEGDMA/BisGMA; Particles of zirconia/silica and colloidal silica	66. 0 wt % 47.0 vol%
RXU	RelyX Ultimate (A3)	3M ESPE, St.Paul, MN	Dual-cure resin cement	Methacrylate monomer, alkaline filler, initiator components, stabilizers, pigments, rheological additives, fluorescence dye	67.0wt % 43.0 vol %

*Bis-GMA* bisphenol-A glycidyl methacrylate, *BDDMA* 1,4-butandioldimethacrylate, *TEGDMA* triethylene glycol dimethacrylate, *UDMA* urethandimethacrylate

**Fig. 1:**
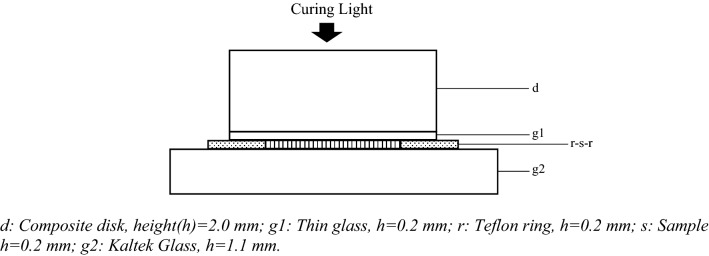
Schematic view of the assembled device for sample polymerization. d: Composite disk, height(*h*) = 2.0 mm; g1: Thin glass, *h* = 0.2 mm; r: Teflon ring, *h* = 0.2 mm; s: Sample *h* = 0.2 mm; g2: Kaltek Glass, *h* = 1.1 mm

Each tested material was placed on a Kaltek Glass (1.1 mm thick) inside a thin Teflon Ring (0.2 mm height and 15.0 mm internal diameter) and covered by another thin glass (0.2 mm thick), to obtain a disk-shape of the material with a diameter of 15.0 mm and a thickness of 0.2 mm (Fig. 1). Samples were divided into two groups and then submitted to the following curing protocols: five samples were cured for 40 s (P1); the other five samples were cured for 5 s, and then, after 20 s, cured again for additional 40 s (P2). The sample size was calculated using MATLAB (version 7.5.0, MathWorks, Natick, MA, USA) and by the analysis of previous studies [8–11]. An Elipar DeepCure S light (3 M Espe, Seefeld, Germany) was adopted, with an irradiance of 1470 mW/cm^2^ ± 20% and a spectrum range between 430 and 480 nm. During curing phases, a polymerized composite disk (Filtek Supreme A3B Plus, 3M) of 2.0 mm of thickness and 25.0 mm of diameter was interposed between the tip of the lamp and the resin cement sample (Fig. 1). The composite disk was removed after each curing phase. The translucency of the composite disk was 10.07; it was calculated using the CIELab space as the difference in colour between the disk as it appeared against the standard white background and as it appeared against the standard black background, according to the following equation:

$${\text{TP}} = \left[ {\left( {{\text{LW}} {-} {\text{LB}}} \right)^{2} + \left( {{\text{aW}} {-} {\text{aB}}} \right)^{2} + \left( {{\text{bW}} {-} {\text{bB}}} \right)^{2} } \right]^{1/2}$$

All the values were evaluated by SpectroShade-Micro (MHT S.p.a., Verona, Italy) on white (W) and black (B) background: L* (lightness, where 100 represents white and 0 represents black), a* (red-green chromatic coordinate) and b* (blue-yellow chromatic coordinate).

The kinetic evaluation of the polymerization process of all samples was performed by a Perkin Elmer Spectrum One NTS FT-NIR spectrometer, operating in the 10000–4000 cm^−1^ spectral range. The NIR spectra of un-polymerized materials were first purchased (T0). Then, samples were cured following the appropriate curing protocol (P1 or P2) and the NIR spectra were collected in continuous mode for 5 min (one every 13 s) using the TimeBase software package (PerkinElmer). The NIR spectra of the same samples were also collected after 1, 2, 7, 14 and 28 days. In the waiting time, samples were stored in dry and dark conditions at room temperature. All the collected spectra were interpolated in the 7000–4000 cm^−1^ spectral region and 2-points baseline corrected.

### DC evaluations

The DC of the resin luting agents was calculated by comparing the height of the following peaks in the un-polymerized and polymerized samples: near 6166 cm^−1^ (RXU, RXV, NX3D and NX3F samples) and near 4744 cm^−1^ (MH and MF samples) (related to the C = C moiety directly involved in the polymerization; bands A); near 5993 cm^−1^ (RXU, RXV, NX3D and NX3F samples) and near 4620 cm^−1^ (MH and MF samples) (Spectrum 10.4 software package, Perkin Elmer). For MH and MF, different NIR peaks were chosen due to the more convoluted spectral profile because their specific composition. For each spectrum, the ratio between the heights of B and A bands was calculated (B/A), and then converted in DC using a calibration curve [12, 13].

### Statistical analysis

After normality and homogeneity evaluations of the data, One-way ANOVA was performed for DC changes, within each protocol. The Tukey HSD test was used for multiple comparisons between groups. Student's t-test was used for the comparisons between the different protocols of the same materials and time points. All tests were performed with *p* < 0.05, using the statistical package Data Analysis in Microsoft Excel 2013 and R Project.

## Results

**Fig. 2:**
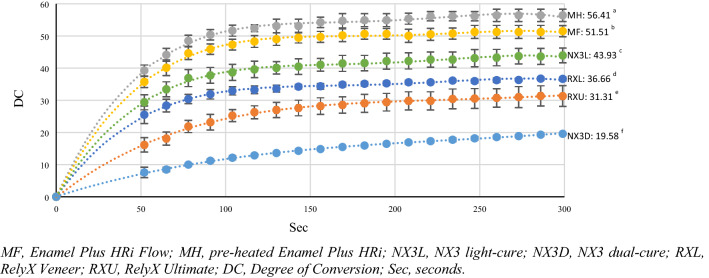
Trend of the Degree of Conversion evaluated in the first 5 min for the tested materials P1. Different superscript letters indicate statistically significant differences (*p* < 0.05)

**Fig. 3:**
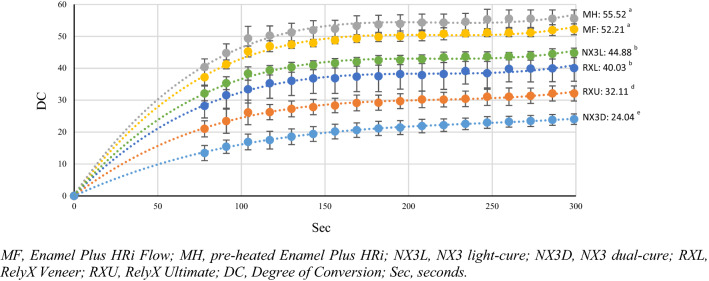
Trend of the Degree of Conversion evaluated in the first 5 min for the tested materials P2. Different superscript letters indicate statistically significant differences (*p* < 0.05)

During the first 5 min, all the tested materials showed an increasing exponential trend for DC values for both P1 and P2 protocols. Moreover, there were significant differences of DC values among all resin luting agents (*p* < 0.05). At the 5-min time point, different DC values were obtained both in relation to the tested materials and the protocols used; in particular, the following significant decreasing order were found: for P1, MH > MF > NX3L > RXL > RXU > NX3D (*p* < 0.05) (Fig. 2); for P2, MH > MF > > NX3L > RXL > RXU > NX3D; however, there were significant differences of DC values among the resin luting agents except between MH and MF, and NX3L and RXL (*p* < 0.05) (Fig. 3). A statistically significant difference between the two protocols was detected only for NX3D (P1, 19.58 ± 0.37 *vs* P2, 24.04 ± 1.64; *p* = 0.004), while MH, MF, NX3L and RXU were not statistically different (*p* < 0.05). The DC values calculated at days 1, 2, 7, 14 and 28 are listed in Table 2. On day 1, MF (73.83 ± 1.61), MH (75.59 ± 2.73) and NX3L (72.73 ± 4.04) had the highest DC in P1 and MF (74.91 ± 0.99), MH (74.16 ± 2.82) in P2, while the lowest DC in both groups was recorded by NX3D (P1: 52.24 ± 0.80; P2: 52.97 ± 1.32). On day 2, MF (78.50 ± 1.01), MH (79.68 ± 1.98) and NX3L (76.76 ± 4.66) had the highest DC in P1, while MF (78.50 ± 1.01) and MH (79.68 ± 1.98) had the highest DC in P2. Conversely, NX3D (58.73 ± 1.29) had the lowest DC in P1, while NX3D (58.19 ± 1.25) and RXU (62.23 ± 5.27) in P2. On day 7, MF, MH, NX3L, RXL showed the highest DC with both the protocols. On day 14, MF, MH, NX3L and RXL had the highest DC in P1 and P2. On day 28, MF, MH, NX3L and RXL had the highest DC in P1 and MH, NX3L and RXL in P2. On days 1, 2, 7, 14 and 28, the effects of P1 and P2 were not statistically different (*p* < 0.05).

**Table 2:** Degree of Conversion evaluated for the tested resin materials after 1, 2, 7, 14 and 28 days

		Day 1	Day 2	Day 7	Day 14	Day 28
MF	P1	73.83 ± 1.61^a^	78.50 ± 1.01^a^	79.41 ± 2.12^ac^	85.97 ± 2.00^a^	89.16 ± 3.24^a^
	P2	74.91 ± 0.99^1^	79.36 ± 0.30^1^	79.66 ± 1.24^1^	87.82 ± 0.97^1^	87.07 ± 1.47^1^
MH	P1	75.59 ± 2.73^a^	79.68 ± 1.98^a^	85.77 ± 2.59^a^	86.64 ± 2.58^a^	92.91 ± 3.13^a^
	P2	74.16 ± 2.82^1^	78.09 ± 6.63^1,2^	85.35 ± 1.06^2^	87.20 ± 1.49^1^	92.75 ± 1.73^2^
NX3L	P1	72.73 ± 4.04^a^	76.76 ± 4.66^a^	86.42 ± 4.57^a^	90.56 ± 3.81^a^	92.27 ± 5.19^a^
	P2	66.42 ± 5.60^2^	76.28 ± 1.95^2^	85.75 ± 2.50^2^	88.83 ± 2.69^1^	93.57 ± 2.17^2^
NX3D	P1	52.24 ± 0.80^b^	58.73 ± 1.29^b^	72.09 ± 1.12^b^	78.08 ± 1.57^b^	82.94 ± 0.85^b^
	P2	52.97 ± 1.32^3^	58.19 ± 1.25^3^	72.85 ± 0.76^3^	78.89 ± 1.06^2^	84.03 ± 0.61^1^
RXL	P1	63.15 ± 1.81^c^	68.37 ± 1.37^c^	86.64 ± 2.52^a^	90.02 ± 3.39^a^	92.51 ± 1.98^a^
	P2	61.98 ± 2.07^2,4^	69.28 ± 7.80^2,4^	81.87 ± 3.06^1,2^	85.56 ± 5.24^1^	89.45 ± 4.12^1,3^
RXU	P1	61.27 ± 6.87^c^	64.56 ± 7.38^c^	73.65 ± 7.88^cb^	79.65 ± 11.42^b^	86.18 ± 10.60^a^
	P2	58.47 ± 4.96^2,4^	62.23 ± 5.27^3,4^	71.06 ± 6.14^3^	79.90 ± 5.09^2^	86.53 ± 4.31^2,3^

Comparisons are valid for each column. Different superscript letters and numbers indicate statistically significant difference (*p* < 0.05). *MF* Enamel Plus HRi Flow (UD3), *MH* Enamel Plus HRi(UD3), *NX3L* NX3 light-cure (Yellow), *NX3D* NX3 dual-cure (Yellow), *RXL* RelyX Veneer (A3), *RXU* RelyX Ultimate (A3)

## Discussion

The longevity of an indirect restoration is directly affected by the resin luting agent [14, 15], and by its DC, whose evaluation may be performed by spectroscopic analysis [12, 16]. In particular, the DC of resin luting agents may influence the chemical and mechanical properties of these materials [17, 18]. Moreover, the DC is a critical factor for biocompatibility and colour stability and it is material dependent [4]: a high DC is essential for long-term functionality, while an inadequate DC can be detrimental to the success of dental restorations [2, 19–21]. In general, the maximum DC reached by resin cements is around 60% and increases after time [22].

In the present study, the kinetic of polymerization of various resin luting agents submitted to different curing protocols was evaluated in the first 5 min and, as further extent, the DC was studied over 28 days to better understand the chemical effects of the tested protocols. A 2.0 mm thick composite disk was used for simulating an indirect restoration. The obtained results showed that ~ 50% of the polymerization reaction of light curing materials occurred during the first 5 min, with the flow resin composite MF and the pre-heated high viscosity resin composite MH showing higher DC values with respect to the other light and dual resin cements. This fact could partly be explained by the higher percentage of filler load of MF and MH (77% and 80%, respectively), and consequently the lower matrix content, with respect to the other tested resin cements. During the polymerization process, the resin cement can create a so-called “uncured chamber”: the monomers start to cure, and the material becomes rigid, trapping the unreacted monomers in the matrix. For this reason, the curing process takes 1–7 days to be completed [23, 24]. As in MF and MH, a low percentage of matrix content, and hence of monomer, could allow a thin layer matrix between monomers and fillers, decreasing the possibility to create uncured chamber. Our results are in agreement with Barceleiro et al. which suggested flowable resin composites are suitable alternative luting agents, when used below a thickness of 2.0 mm or less [25].

In summary, in the first 2 days, MF, MH and NX3L showed significantly higher DC compared with the other materials. After 7 and 14 days, DC of RXL resulted similar to that of MF, MH and NX3L, without significant difference, and the DC of dual-cure resin cements were significantly higher than that of light-cure resin cements. Although the dual cement is chemically activated, the low contribution of light curing is not enough to reach a high DC, thus requiring more time to complete the polymerization process and reach a high value. Another possible explanation could be the suboptimal concentration of curing inhibitors [26, 27]. Inhibitors can be added to resin cement to increase the material manipulation and clinical working time [28]. Although the light curing materials also contain inhibitors, the concentration is relatively lower than the dual-cured materials. Consequently, a good balance between initiators and inhibitors is essential for clinical uses [28].

Our results agree with another study evaluating DC of resin luting agents when used under ceramic materials instead of composite. Filho et al. found that resin cements present low DC when the materials are dually activated through 2.0 mm of reinforced ceramic materials with translucency equal to or less than that of IPS Empress [5]. The translucency of IPS Empress was 10.37 with 2.3 mm of thickness [29]. In our study, the translucency of the composite disk used during the polymerisation phases was 10.07 and then it is less translucent than IPS Empress. Moreover, the translucency of our composite disk is lower than IPS e.max Press, IPS e.max CAD and Zirconia materials at different thickness [30]. Therefore, our result could relate to indirect restorative materials with a translucency equal or higher than 10.

Regarding the long-term evaluations, the tested protocols were not statistically different. Although different curing modes have been described in the literature, no data exists about DC effect of such “step luting” protocol P2, with a 28-day long evaluation. The P2 can be considered a modify pulse-delay curing, where the polymerization is initiated by a short flash of light followed by a waiting time of several minutes before the final cure is performed. However, in our P2, only 20 s were between the pre-curing phase (5 s) and the final curing phase (40 s).

In the tested P1, the total irradiance was 58,800 mW/cm^2^ (1470 mW/cm^2^ times 40 s) and no pre-curing phase was applied. In the tested P2 the total irradiance was 7350 mW/cm^2^ for the pre-cure phase (1470 mW/cm^2^ times 5 s), and 58,800 mW/cm^2^ for the final curing (1470 mW/cm^2^ times 40 s). No difference was noted between the tested curing protocols of P1 and P2 at 1, 2, 7, 14, 28 days.

In the P2 protocol, the low energy (7350 mW/cm^2^) of the first 5 s of curing could initiate the conversion of the resin luting agent to a semi-solid state and could allow the indirect restoration to be fixed to the tooth, not affecting the chemical stability of the material. This finding is in partial agreement with Asmussen et al., although they used different pre-cured phases of 10, 20 and 40 s followed by 20 s of final curing, the final DC was not influenced by the low energy density of the pre-cure phase (from 250–16000 mW/cm^2^) which is in accordance with our precured energy density of P2 (7350 mW/cm^2^) [31]. However, in their study the final curing phase was immediately after the pre-curing phase, without the 20 s waiting time. At a high energy density of the pre-curing phase, the polymerization would proceed at a normal and high rate. While a pre-curing phase at low energy density could start the polymerization process by the formation of limited oligomers, building up discontinuous foci of polymerized material and creating microgel regions [32, 33]. This kind of microgel state would allow the clinician to easily remove the excesses when the material is starting to become hard.

Despite the lack of physical and evaluations about adhesion, we can conclude that the light curing resin cements achieved a clinically acceptable DC after 5 min. Furthermore, all the resin luting agents reached more than 50% DC after 1 day. Over the period, the light-cure luting cements had the higher DC values than the dual cured ones. In conclusion, we can reject the first null hypothesis, because our results suggest that all the tested materials did not reach the same DC, and accept the second one because of the two different curing protocols seem to not influence DC values, also over a long time. Then, the clinician can safely use the tested “step luting” protocol (5 s + 40 s) to lute the indirect restoration, simplifying the removal of cement excesses, in particular in the interdental space.
